# Evaluation of the Effect of Silver Diamine Fluoride Treatment with and without Glutathione and Potassium Iodide on the Micro-shear Bond Strength of CR to Primary Tooth Dentin -

**DOI:** 10.31661/gmj.vi.3898

**Published:** 2025-11-29

**Authors:** Masoumeh Khataminia, Akramsadat Emami, Leila Basir

**Affiliations:** ^1^ Department of Pediatric Dentistry, School of Dentistry, Ahvaz Jundishapur University of Medical Sciences, Ahvaz, Iran

**Keywords:** Silver Diamine Fluoride, Glutathione, Potassium Iodide, Micro-shear Bond Strength, Composite Resin

## Abstract

**Background:**

Silver diamine fluoride (SDF) is widely employed to halt caries in juvenile or uncooperative children due to its antimicrobial and remineralizing properties. Despite its effectiveness, SDF causes dark discoloration of treated dentin, raising esthetic concerns. Potassium iodide (KI) and glutathione (GSH) have been proposed as adjuncts to reduce discoloration; however, their influence on subsequent adhesive bonding to primary dentin remains unclear. This study evaluated and compared the micro-shear bond strength (μSBS) of composite resin (CR) to demineralized primary tooth dentin treated with SDF alone, SDF combined with KI or GSH, and untreated dentin.

**Materials and Methods:**

Fifty-two sound primary teeth were prepared to obtain flat mid-coronal dentin surfaces and artificially demineralized. Specimens were randomly assigned to four groups (n=13): control (no SDF), SDF, SDF+KI, and SDF+20% GSH. After pretreatment, all samples underwent phosphoric acid etching, application of a total-etch adhesive, and restoration with CR using standardized molds. μSBS was measured with a universal testing machine operating at a crosshead displacement rate of 1 mm/min. Statistical evaluation of the data was conducted via one-way analysis of variance (ANOVA) followed by Tukey’s post-hoc comparisons, with the significance level set at 0.05.

**Results:**

SDF-treated groups demonstrated significantly higher μSBS values compared with the control group (P0.001). Mean bond strengths were highest for SDF alone, followed closely by SDF+KI and SDF+GSH. The data revealed no meaningful divergence in the outcomes of the three SDF-based treatments.

**Conclusion:**

Pretreatment of demineralized primary dentin with SDF significantly enhanced CR bond strength. The addition of potassium iodide or glutathione did not adversely affect bonding performance, suggesting these agents may be used to reduce discoloration without compromising adhesive outcomes.

## Introduction

Silver Diamine Fluoride (SDF) is a colorless substance containing fluoride ions that helps reduce the demineralization of tooth tissue. It is also used to arrest root caries, control dental decay in children, prevent secondary caries formation, treat infected root canals, and desensitize teeth [1]. When the child’s age is young for restorative treatment and cooperation is limited, SDF is used to slow down and halt the progression of caries [2]. In addition to arresting dentinal caries, SDF forms a hard, black, and impermeable layer on the tooth, and precipitates of silver phosphate deposits upon the dental substrate, which is not easily soluble [3][4]. The black color caused by SDF in esthetic areas can lead to concerns among patients and parents. To improve patient satisfaction and reduce this discoloration, the application of a potassium iodide solution has been suggested immediately after SDF application [5]. Potassium iodide reacts with the excess silver ions and forms a cream/yellowish-white precipitate of silver iodide, which is cream-colored, thereby masking the discoloration of SDF [6][7]. Glutathione forms a layer around the silver particles and reduces the rate of spread and aggregation of silver particles, which may decrease the speed of color changes on the surface of the tooth covered with SDF [8].

Dentin exists as a mineralized biological matrix—a composite of both organic and inorganic phases, permeated by fluid and structured as a specialized connective tissue [9][10]. The use of fluoride to prevent dental caries, remineralize enamel caries, and even arrest dentinal caries has been confirmed [11]. According to the results of previous studies, the superiority of self-etch adhesives (including universal adhesives) compared to traditional acid etch and rinse adhesives for bonding to sound dentin has been reported in some contexts [12]. Studies have also shown that SDF-based anti-caries agents (including nanoparticles) leaves the composite resin's micro-shear bond strength uncompromised, whether on intact or demineralized enamel [13]. In a separate investigation examining SDF and its influence on adhesion, researchers prepared human coronal middle dentin specimens and subjected them to a three-minute application of either distilled water (serving as the control), 3.8% SDF, or 38% SDF. Following this pre-treatment, a two-step self-etch adhesive system and CR were meticulously placed in accordance with the manufacturer’s protocols. The bonded assemblies subsequently underwent thermocycling across three intervals: 0, 5000, and 10,000 cycles [14]. Several studies have investigated the positive effects of glutathione and potassium iodide on reducing the discoloration of SDF, and since restorative treatment with composite is performed after the patient becomes older and more cooperative, the present study aimed to evaluate and compare the μSBS of CR to primary tooth dentin treated with SDF, with or without potassium iodide and glutathione, and untreated dentin (control).

## Materials and Methods

### Specimen Preparation

The present study was an experimental interventional laboratory study that utilized sound primary teeth with no restorations or caries, obtained from clinics and private practices in Ahvaz. The teeth were immediately stored in a 0.1% thymol solution (Merck, Darmstadt, Germany) after extraction. The teeth were mounted in self-cure acrylic resin (Acropars, Marlic Co., Tehran, Iran) with the cementoenamel junction (CEJ) positioned 1 mm above the acrylic surface to facilitate handling and movement. A low-speed diamond saw, under constant water irrigation, was employed to resect the occlusal enamel, thereby revealing a uniform mid-coronal dentin plane. This exposed surface was subsequently refined via 10-second polishing with 600-grit silicon carbide paper under a stream of water. To generate simulated demineralized dentin, specimens were immersed in a demineralizing agent (pH 4.4, formulated with 50 mM acetate buffer, 2.2 mM potassium dihydrogen phosphate, and 2.2 mM calcium chloride); all chemicals from Merck, Darmstadt, Germany) for 7 days at 37°C. Each tooth provided one test specimen.

### Group Allocation

Sample size was determined to be 52 specimens in total. From the total pool of specimens, a randomized allocation established four distinct experimental cohorts (n=13 each) according to the SDF-based pretreatment applied prior to bonding:

1. Control group (no SDF pretreatment)

2. SDF group

3. SDF + potassium iodide (SDF+KI) group

4. SDF + 20% glutathione (SDF+GSH) group

### Pretreatment Procedures

All SDF applications and bonding procedures were performed according to the manufacturers’ instructions. In the control group, the demineralized dentin surface was rinsed with normal saline (Samen Pharmaceutical Co., Mashhad, Iran) only. In the SDF group, 38% silver diamine fluoride (Cobalt, India) was applied to the dentin surface using a microbrush for 1 minute, followed by rinsing with distilled water for 30 seconds. In the SDF+KI group, SDF was applied as described, immediately followed by application of potassium iodide with a microbrush until a creamy white precipitate formed, after which the surface was rinsed with distilled water for 30 seconds. In the SDF+GSH group, a freshly prepared mixture of 38% SDF and 20% glutathione was applied as a single solution using a microbrush for 1 minute, followed by rinsing with distilled water for 30 seconds [15].

### Bonding and Restoration Procedure

A standardized bonding protocol was then implemented across all groups. The dentin surface was conditioned with 35% phosphoric acid for 15 seconds, thoroughly rinsed with distilled water for 10 seconds, and subsequently dried with a gentle air stream. Two uniform layers of the total-etch adhesive (Single Bond 2, 3M ESPE) were then applied, lightly air-dispersed, and polymerized for 15 seconds using an LED light-curing unit (Kerr, USA) emitting an intensity of 900 mW/cm².

To define the bonding site, a cylindrical silicone mold (4 mm internal diameter, 3 mm height) was placed on the prepared surface prior to adhesive polymerization. The mold cavity was incrementally filled with CR (Filtek™ Z250, 3M ESPE, St. Paul, MN, USA) in two layers, each receiving 40 seconds of light activation.

### μSBS Testing

μSBS testing was performed using a universal testing machine (Zwick/Roell, Ulm, Germany). The shear load was applied with a blade positioned at the dentin-composite interface, parallel to the flat dentin surface and perpendicular to the long axis of the composite cylinder. A crosshead speed of 1 mm/min was used until failure occurred. Specimens that debonded prematurely during handling were assigned a bond strength value of 0 MPa. Bond strength values (in MPa) were calculated by dividing the maximum load at failure by the bonding area (12.56 mm²) [12].

### Statistical Analysis

Quantitative outcomes are presented as mean ± standard deviation. The assumption of normality was verified through the Kolmogorov-Smirnov test. Intergroup comparisons for mean μSBS values were conducted via one-way analysis of variance (ANOVA), with subsequent post-hoc analysis performed using Tukey's test. Qualitative observations, including failure mode distribution, were subjected to Chi-square analysis. A predetermined alpha level of 0.05 defined statistical significance. All computational procedures were executed with SPSS software (version 22.0).

## Results

**Figure-1:**
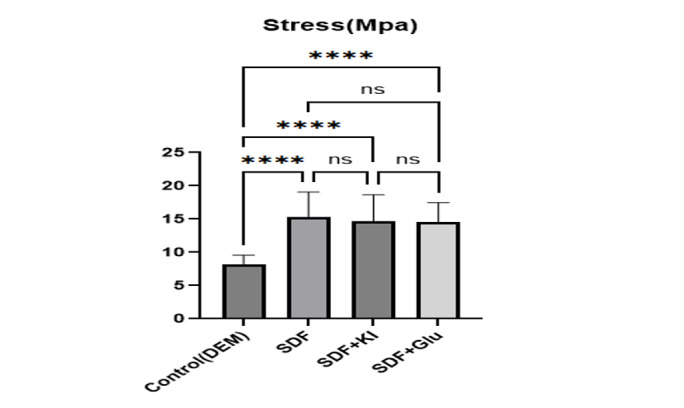
Figure 1. ΜSBSof CR to dentin treated with three methods: SDF, SDF + potassium iodide, SDF + 20% glutathione, and the control group

**Table:** Table1. μSBS of CR to Dentin

**Group**	**Mean ± SD (MPa) **	**Range (Min-Max, MPa) **	**Mean Difference vs. Control (MPa) **	P-value vs. Control*	**P-value vs. SDF† **	**P-value vs. SDF+KI† **
**Control**	8.24 ± 1.31	6.66 - 11.52	—	—	< 0.001	< 0.001
**SDF**	15.34 ± 3.72	10.23 - 22.47	7.09	< 0.001	—	0.951
**SDF + Potassium Iodide (SDF+KI) **	14.68 ± 3.96	11.18 - 25.49	6.43	< 0.001	0.951	—
**SDF + 20% Glutathione (SDF+GSH) **	14.51 ± 2.97	9.21 - 18.18	6.27	< 0.001	0.910	0.999

The μTBS of CR to dentin was significantly influenced by the application of SDF-based treatments compared to the untreated control group (one-way ANOVA: F=14.27, P<0.001). The control group exhibited the lowest bond strength, with a mean of 8.24 ± 1.31 MPa (range: 6.66-11.52 MPa). In contrast, all three SDF-treated groups demonstrated substantially higher bond strengths: SDF alone achieved the highest mean of 15.34 ± 3.72 MPa (range: 10.23-22.47 MPa), followed by SDF + potassium iodide (SDF+KI) at 14.68 ± 3.96 MPa (range: 11.18-25.49 MPa), and SDF + 20% glutathione (SDF+GSH) at 14.51 ± 2.97 MPa (range: 9.21-18.18 MPa). Post-hoc Tukey pairwise comparisons confirmed that each SDF-based treatment yielded significantly higher μSBS than the control (P<0.001 for all three comparisons; mean differences: 7.09 MPa for SDF vs. control, 6.43 MPa for SDF+KI vs. control, and 6.27 MPa for SDF+GSH vs. control), whereas no significant differences were observed among the three SDF-treated groups (P=0.951 for SDF vs. SDF+KI, P=0.910 for SDF vs. SDF+GSH, and P=0.999 for SDF+KI vs. SDF+GSH), as also illustrated in Figure-1.

## Discussion

Recent research has highlighted SDF as a promising agent for arresting and preventing dental caries [2]. Its effectiveness is attributed to the combined remineralizing and antibacterial properties of fluoride and silver ions at specific concentrations [16]. A persistent challenge to its widespread clinical adoption, however, is the permanent, dark discoloration of the arrested carious lesion [17]. In response, multiple strategies have been explored to mitigate this undesirable staining. These include substituting SDF with alternative agents like fluoride varnish or ammonium hexafluorosilicate [18][19], employing silver nanoparticles [20][21], and applying post-treatment solutions such as saturated potassium iodide [22] or 20% glutathione [23][24].

The application of potassium iodide (KI) immediately after SDF treatment is a notable method; it reacts with free silver ions to form a cream-colored silver iodide precipitate, thereby circumventing the formation of the dark silver phosphate [5][24]. As for glutathione, a ubiquitous cellular non-protein thiol with antioxidant properties, it possesses a high affinity for metal ions. Its mechanism involves chelating silver particles, enhancing their solubility in aqueous environments and integrating them into complex biological systems [6].

In this investigation, μSBS testing was employed to assess the adhesive performance of the specimens. Although μTBS testing is frequently regarded as the benchmark method, shear testing offers distinct advantages when evaluating the impact of material interventions on adhesion; notably, specimen preparation is markedly less time-intensive [12].

Statistical analysis revealed a significant variation in μSBS between the control group and dentin pre-treated with SDF alone, SDF followed by potassium iodide, and SDF followed by 20% glutathione. The control group demonstrated the lowest mean bond strength. No statistically significant divergence in μSBS was detected among the three experimental SDF-based treatment groups. This outcome finds support in the work of Wu et al., who reported that the mechanical bonding properties of CR to dentin treated with 38% SDF did not differ significantly from those to untreated dentin, suggesting that SDF application does not detrimentally affect the integrity of an overlying resin restoration [25]. In the SDF-treated groups of the present study, failure was frequently localized to the adhesive layer, implying that the bond between the adhesive and the SDF-conditioned dentin substrate may, in fact, be robust. These observations align with the findings of Selvaraj et al., whose failure mode analysis similarly identified adhesive failure as predominant across all groups, followed by cohesive failure within the resin or dentin [26]. Furthermore, their transmission electron microscopy (TEM) evaluation of the hybrid layer indicated that a combined SDF/KI pre-treatment effectively reduced nanoleakage at the resin-dentin interface without compromising bond strength, irrespective of the adhesive system used. This suggests that such a pre-treatment protocol may not only preserve but potentially enhance the quality of the adhesive resin bond.

A review of prior investigations reveals a complex relationship between SDF application and subsequent adhesion. Frohlich et al., in their analysis of bond strength for glass ionomer cements and adhesive systems, reported no significant difference between control specimens and those treated with unwashed SDF in both aggregate and subgroup analyses, concluding that SDF does not compromise the bond of glass ionomer to dentin [27]. Similarly, Jaqueline Costa Favaro et al. affirmed that anti-caries agents, including SDF, do not adversely affect the micro-shear bond strength of CR on either sound or demineralized enamel [13]. Conversely, Lutgen et al. observed that the impact of SDF on microtensile and shear bond strengths was protocol-dependent, noting a generally negative effect that was mitigated when specimens were rinsed following SDF application [28]. This evidence directly informed the methodology of the current study, where a washing step was incorporated post-SDF application. The consensus from multiple studies is that the adhesive outcome is critically dependent on the specific application protocol [27][29]. Supporting this, el-Ghamrawy et al. employed a wash step to eliminate excess silver precipitates and fluoride ions from dentinal tubules, aiming to preserve therapeutic efficacy while minimizing interference with the bonding substrate [29][30]. The rationale is that without such removal, these residual deposits may impede proper resin infiltration into the dentin, ultimately degrading the bond integrity [27].

The findings of Markham et al., indicating a reduction in CR bond stability on SDF-treated enamel and dentin when using universal adhesives, stand in contrast to the results presented here [31]. This divergence may be attributed to the distinct adhesive systems employed—specifically, the universal bonding agent in their study versus the total-etch system utilized in the present investigation. Further contextualizing this variability, Koizumi et al. evaluated the effect of unrinsed SDF/KI application on the bond strength of various adhesive strategies, including etch-and-rinse, two-step self-etch, and all-in-one adhesives, alongside resin-modified glass ionomer [32]. They reported a significant decline in bond strength for the adhesive groups, with SEM analysis attributing this to SDF deposition on the dentin surface. Notably, the total-etch and glass ionomer groups were less affected. The protocol in the current study, employing a total-etch system and, critically, incorporating a rinse after KI application, likely accounts for the observed discrepancy with Koizumi’s outcomes. This shows a key methodological challenge highlighted by Jiang et al.: the absence of a standardized protocol for specimen preparation and SDF application precludes definitive conclusions regarding SDF’s impact on dentin bonding [29]. The observed inconsistencies across studies are thus plausibly rooted in this procedural heterogeneity.

## Conclusion

Based on the present findings, it can be inferred that the administration of SDF, a mixture of SDF and glutathione, and SDF treatment combined with potassium iodide before composite restoration significantly increases bond strength.

## Conflict of Interest

None.
